# Effective Data Sharing as a Conduit for Advancing Medical Product Development

**DOI:** 10.1007/s43441-020-00255-8

**Published:** 2021-01-04

**Authors:** Stephen R. Karpen, J. Kael White, Ariana P. Mullin, Inish O’Doherty, Lynn D. Hudson, Klaus Romero, Sudhir Sivakumaran, Diane Stephenson, Emily C. Turner, Jane Larkindale

**Affiliations:** Critical Path Institute, 1730 E. River Rd. Ste 200, Tucson, AZ 85718 USA

**Keywords:** Data, Data-sharing, Regulatory science, Drug development tools, Product development, Public–private partnerships

## Abstract

**Introduction:**

Patient-level data sharing has the potential to significantly impact the lives of patients by optimizing and improving the medical product development process. In the product development setting, successful data sharing is defined as data sharing that is actionable and facilitates decision making during the development and review of medical products. This often occurs through the creation of new product development tools or methodologies, such as novel clinical trial design and enrichment strategies, predictive pre-clinical and clinical models, clinical trial simulation tools, biomarkers, and clinical outcomes assessments, and more.

**Methods:**

To be successful, extensive partnerships must be established between all relevant stakeholders, including industry, academia, research institutes and societies, patient-advocacy groups, and governmental agencies, and a neutral third-party convening organization that can provide a pre-competitive space for data sharing to occur.

**Conclusions:**

Data sharing focused on identified regulatory deliverables that improve the medical product development process encounters significant challenges that are not seen with data sharing aimed at advancing clinical decision making and requires the commitment of all stakeholders. Regulatory data sharing challenges and solutions, as well as multiple examples of previous successful data sharing initiatives are presented and discussed in the context of medical product development.

## Patient-Level Data Sharing Facilitates Innovation in Medical Product Development

Patient-level data sharing is fundamental to the advancement of science and improvement of public health. A previous *JAMA* editorial [1] describes the ethical and scientific imperative of data sharing, pressing the importance of sharing data to verify a study’s original analyses and to aid in hypothesis generation. These, and other benefits of data sharing, have primarily been focused on advancing basic science and improving clinical care decision making. Unfortunately, many of the benefits of data sharing in the clinical context often cannot directly translate to advances in product development. To meaningfully leverage data sharing to benefit product development, the regulatory setting must be a primary focus. When successful, sharing data allows the process of developing novel therapies to become more efficient, allowing timelier availability of life changing therapies for patients in need.

In the patient care setting, data sharing has led to improved decision-making through several means, including, aiding in the development of clinical decision support tools, extending learnings from single organizations to an entire field, and ensuring treatment guidelines are up-to-date, standardized, and well informed [2, 3]. Patients directly benefit through optimized use of already approved therapies. This has been particularly exemplified during the COVID-19 pandemic, where rapid sharing of data between hospital centers and from clinical trials has resulted in improvements in clinical care. For example, the CURE-ID application [4], developed in 2013 as a collaboration between the US Food and Drug Administration (FDA) and the National Center for Advancing Translational Sciences (NCATS), is an internet-based repository that lets the clinical and research community share de-identified data regarding novel uses of exiting drugs for difficult to treat or emerging infectious diseases. CURE-ID currently includes thousands of case reports of patients who have been treated for COVID-19 and an aggregated list of COVID-19 trials from clinicaltrials.gov. The CURE-ID application allows clinicians to access the entirety of this data in one unified place to make more informed treatment decisions for their patients. In addition to facilitating this kind of clinical data sharing for COVID-19, CURE-ID includes shared data to inform clinical decision making for over 325 infectious diseases.

When considering medical product development, patients benefit as data sharing can reinvigorate drug development in therapeutic areas deprioritized by industry and help overcome scientific challenges, leading to accelerated development of novel therapies. However, the evidentiary rigor and data standards required for developing new medical products differ significantly from those required to inform clinical practice, adding complexity to the goal of realizing the potential of data sharing to catalyze medical product development.

Medical product development is multifaceted, complex, and expensive, with an inherently high degree of uncertainty. As a single product advances through the development process, the costs and risks of failure increase significantly [5]. Advances in basic science and technology have led to more potential new therapies under development, but the time from discovery to approval remains long, costs remain high, and overall success rates (i.e. rates of new products that receive marketing authorization) remain low. A recent report by Wouters et al. [6] estimates a mean expenditure of $1.3 billion (2018 dollars) to develop a novel therapeutic, when accounting for failed trials and other cost considerations. Data sharing initiatives, particularly those aimed at optimizing clinical trials, reduce time and costs required to bring safe and effective therapies to patients, improving public health. Effective sharing of patient-level data can support innovation throughout the medical product development process, here we focus on the use of such data to develop novel tools and methodologies that optimize this process.

## Sharing Patient-Level Data Drives Development of Innovative Tools and Methodologies

In its landmark 2004 report, “Innovation/Stagnation: Challenge and Opportunity on the Critical Path to New Medical Products,” FDA states “a new product development tool kit, containing powerful new scientific and technical methods such as animal or computer-based predictive models, biomarkers for safety and effectiveness, and new clinical evaluation techniques” is an essential and urgent need to ensure patients continue to have access to new, safer, and more effective therapies [7]. Significant progress has been made since the 2004 report, however better tools and methodologies to improve product development remain primary goals of legislative actions, such as the 2017 reauthorization of the Prescription Drug User Fee Act (PDUFA VI) and the twenty-first Century Cures Act (Cures Act), which, among other goals, seeks to bring innovation and efficiency to medical product development. The European Medicines Agency (EMA) has also indicated the need for novel methodologies or tools for better development of medicinal products is a priority of the agency [8].

Collectively, these tools are referred to as drug development tools (DDTs) or medical device development tools (MDDTs). DDTs/MDDTs are defined as methods, materials, or measures that can potentially facilitate the drug or device development process [9, 10]. DDTs/MDDTs can include novel clinical trial designs (e.g., umbrella or platform trials) and enrichment strategies, predictive pre-clinical and clinical models, clinical trial simulation tools, novel biomarkers and clinical outcomes assessments, and more. In general, the overall goal of these tools is to reduce the inherent uncertainty in the decisions that developers make during the product development process. For example, these decisions may include whether to move an investigational product from one phase of development to the next, whether a study endpoint is appropriate for a given population, or whether pre-clinical safety will be maintained in human trials. Uncertainty in these decisions, and many others, can lead to significant increases in the time, risk, and costs required to bring new medications to patients. By reducing uncertainty through the development and use of DDTs/MDDTs, decision making during product development is more informed, ultimately resulting in timelier patient access to improved therapies.

**Figure 1.**
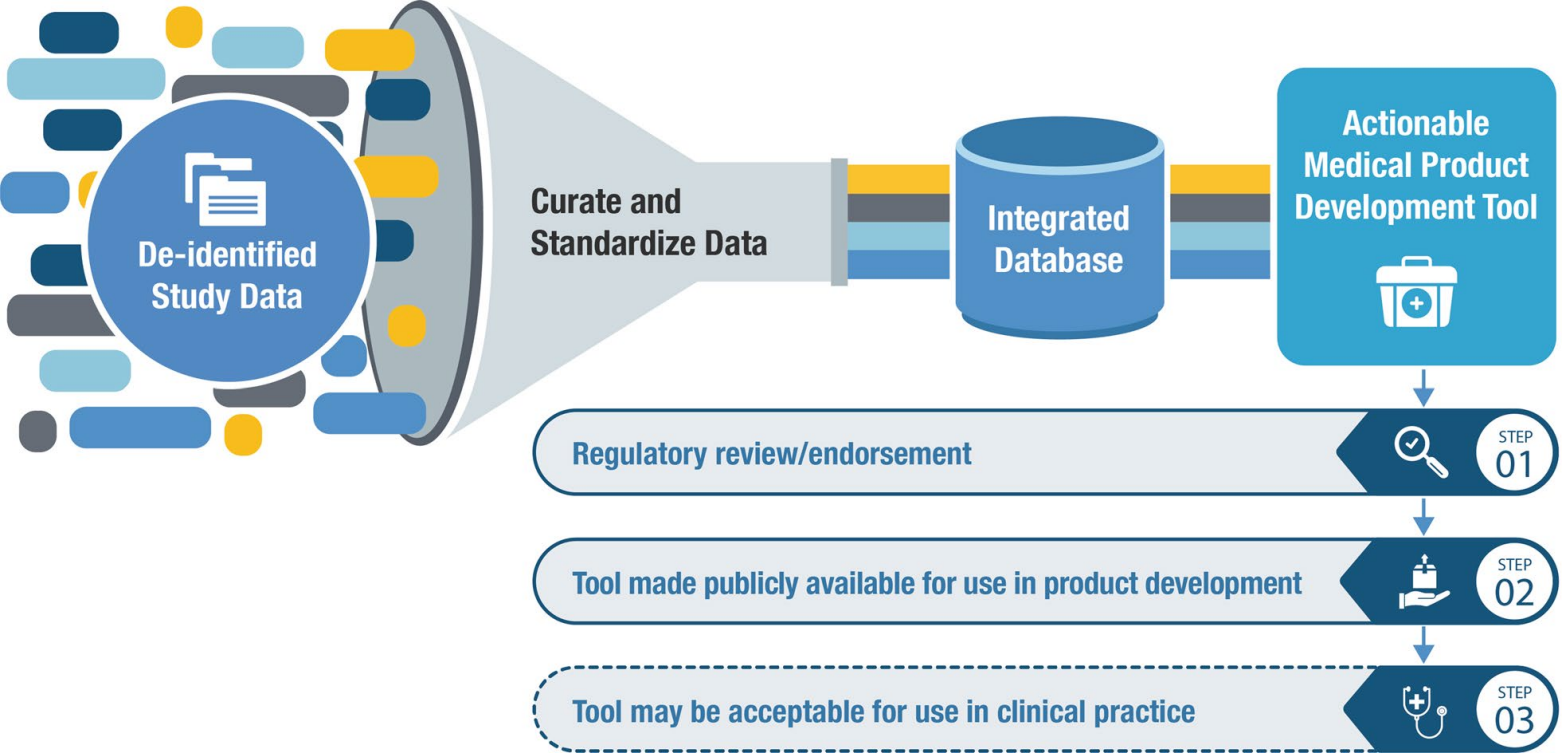
Overview of Patient Level Data Sharing to Develop Medical Product Development Tools.

Once developed, these tools can be submitted to regulatory authorities through various established pathways [10–12] to seek endorsement supporting their use in product development. Regulatory endorsement provides developers with confidence in using a tool and requires tools be publicly available, encouraging their broad implementation in development programs. The evidentiary standards for regulatory endorsement are high, and significant data is typically required to support a tool’s use. When tools are developed collectively through collaborations and made publicly available for widespread use, the individual burden of tool development is reduced, allowing simultaneous benefits to product developers, regulators, and most importantly, patients. Importantly, many tools endorsed for use in the product development setting can also be used downstream to inform and improve clinical care (Fig. 1).

The totality of evidence required to support the use of a tool is dependent on its intended application or context-of-use, but frequently requires patient-level data from multiple independent sources or studies, potentially including data from real-world sources. Successful data sharing, in this context defined as data sharing that facilitates decisive action in medical product development programs (i.e. actionable data sharing), therefore relies on extensive partnerships between industry, academia, research institutes and societies, patient-advocacy groups, and governmental agencies. As suggested by FDA’s Critical Path Initiative, public–private partnerships (PPPs) or consortia are conduits to maximizing the generation of solutions that expedite product development. PPPs or consortia, defined by FDA as “collaborative groups managed by a convening or coordinating organization involving multiple stakeholder organizations including at least one non-profit or 501(c)(3) organization (e.g., academia, government, or foundation) and at least one for-profit organization (e.g., pharmaceutical, biotechnology, or medical device company),” [13] provide safe harbors for individual organizations to share data and while minimizing concerns for losing competitive advantages. Tools created by PPPs are often submitted for regulatory endorsement and made publicly available, further expanding their impact.

Meaningful steps to make patient-level data from clinical trials and other high-quality studies widely available have already been taken in some therapeutic areas, most notably in the field of oncology. For example, Project Data Sphere has successfully housed more than 150 datasets from over 100,000 cancer patients, and provides community access to these data [14]. Data have been accessed nearly 20,000 times and have supported 81 publications to date. The data have also supported the development of multiple new tools that will aid product development in different cancers, including models of the relationship between prostate-specific antigen and survival in metastatic castrate-resistant prostate cancer [15], and the use of the baseline albumin-bilirubin (ALBI) score as a prognostic biomarker in patients with hepatocellular carcinoma [16]. Oncology, long touted as a leading example of innovation in product development, has built its success on long-standing emphasis on collaboration and data sharing across cancer types through national oncology initiatives, such as those of the Oncology Center for Excellence and the National Cancer Institute.

Recently, other therapeutic areas, particularly in rare diseases, have launched major initiatives to encourage similar advances in data sharing. For example, the Asia Pacific Economic Cooperation Rare Disease Registry and Analytics Platform (APEC RD-RAP) and the Rare Disease Cures Accelerator-Data and Analytics Platform (RDCA-DAP) are being developed to improve product development across all rare diseases. There are many examples of disease-specific patient-level databases, such as the Pooled Resource Open-Access ALS Clinical Trials Database (PRO-ACT) [17] for amyotrophic lateral sclerosis, and databases in Friedreich’s ataxia [18], multiple sclerosis [19], polycystic kidney disease [20], tuberculosis [21], and others.

An important component of the precompetitive environment provided by PPPs is the possibility for involvement of the regulatory agencies. PPPs may request a scientific liaison from FDA, allowing for close and active regulator engagement with the scientific community developing a new tool and improved alignment of the PPP’s deliverables with Agency priorities areas and PDUFA and Cures Act goals. Currently, FDA participates in 45 PPPs, many already delivering tools that have improved medical product development [13].

## Examples of Development of DDTs Based on Successful Data Sharing

Patient-level data sharing can help create solutions to many challenges in the medical product development process by generating and making new knowledge widely accessible to the community. When successful, learnings actionably reduce risk and increase confidence in regulatory decisions on specific products. With only 59% of drug development programs that enter Phase 3 clinical trials ultimately obtaining FDA approval [22], the ability to halt development programs prior to initiating late-stage trials reduces costs, allows reallocation of resources to products more likely to succeed, and reduces harm to patients by decreasing exposure to investigational products. When investigational products reach late stage development, optimizing clinical trials reduces the time, size, and cost of these trials, and can improve confidence in the trial results. Examples of how successful patient-level data sharing has impacted several aspects of medical product development are discussed here and in Table 1.

**Table 1.** Examples of the Value of Analyzing Shared Patient-level Data.

Drug Development Challenge	Solution	Disease	Study Group/Consortium	RCT/Non-RCT	Citation(s)
Understanding placebo response	Drug disease trial model including placebo response	Alzheimer’s disease Huntington’s disease^a^ Parkinson’s disease^a^ Duchenne muscular dystrophy^a^	CPAD HD-RSC CPP D-RSC	Both	Romero et al. [36]
Assessment of disease progression across multiple endpoints	Stage specific outcome measures based on quantitative disease progression model	Duchenne muscular dystrophy^a^	D-RSC	Both	Conrado et al. [35]
Prediction of clinical diagnosis for disease prevention	Quantification of disease progression as a function of biomarker status prior to diagnosis	Type I diabetes	T1D	Both	Rasi [25]
Prediction of rate of disease progression	Quantification of disease progression as a function of baseline characteristics	Autosomal dominant polycystic kidney disease Duchenne muscular dystrophy Amyotrophic lateral sclerosis	Academic/industry CTAP PRO-ACT	Both	McEwan et al. (2018), Mercuri et al. (2016), Goemans et al. (2016), Zandona et al. (2019), Tang et al. (2019), Karanevitch et al. (2018), Daghlas et al. (2017)
Disease prognosis and subject selection	Enrichment biomarker, model-based biomarker qualification Stratification of patient populations	Polycystic kidney disease Alzheimer’s disease Parkinson’s disease Amyotrophic lateral sclerosis	PKD CPAD CPP PRO-ACT	Both Both	Hill et al. Conrado et al. Pharsight/JD Berry et al., Ong et al., Taylor et al
Trial design optimization	Clinical trial simulation tool based on drug disease trial model Analysis of the value of use of historical controls	Alzheimer’s Disease Parkinson’s Disease Duchenne muscular dystrophy Huntington’s disease^a^ Amyotrophic lateral sclerosis	CPAD CPP D-RSC HD-RSC PRO-ACT	Both Both	Neville Romero Conrado Schoenfeld et al
Validation of outcome assessments	Qualification of outcome measure Validation of outcome measures	MS Kidney transplantation^a^ Crohn’s Disease Amyotrophic lateral sclerosis	MSOAC TTC Academic PRO-ACT	Both Clinical trials Both	Rudick et al Thia et al., (2011) Van Eijk et al. (2018)
Validation of biomarkers	Qualification of prognostic biomarker	Polycystic kidney disease Parkinson’s Disease	PKDOC CPP	Non-RCT Both	Perrone et al. (2017) Stephenson et al. (2018)

*CPAD* Critical Path for Alzheimer’s Disease, *CPP* Critical Path for Parkinson’s, *CTAP* Collaborative Trajectory Analysis Project, *D-RSC* Duchenne Regulatory Science Consortium, *HD-RSC* Huntington’s Disease Regulatory Science Consortium, *MSOAC* Multiple Sclerosis Outcome Assessments Consortium, *PKDOC* Polycystic Kidney Disease Outcomes Consortium, *PRO-ACT* Pooled Resource Open-Access ALS Clinical Trials Database, *T1D* Type 1 Diabetes Consortium

^a^Ongoing efforts

### Disease Prognosis and Subject Selection

Onset and rate of progression of chronic progressive neurodegenerative diseases, such as Parkinson’s disease (PD) are heterogeneous between patients. Potential therapies are more likely to slow progression than reverse damage and would ideally be initiated as early as possible in the course of disease. Poor ability to select patients with early disease for clinical trials has resulted in mixed study populations that do not fully reflect the target population. Trials must, therefore, include a larger sample size to adequately detect drug effects, adding inefficiency. To meet this need, the Critical Path for Parkinson’s (CPP) used an integrated patient-level database consisting of data from 672 patients from 2 independent clinical studies to build models that describe the use of dopamine transporter (DAT) neuroimaging as an enrichment biomarker for early stages of PD. This biomarker has been qualified through EMA’s Qualification of Novel Methodologies for use in Clinical Trials program [23]. The model allows drug sponsors to design trials of similar power with reduced sample size, relative to the same trial design without the use DAT imaging. This tool may be used to select optimized patient populations for trials, reduce sample size in trials, and accelerate the rate at which informative trials can be completed [24].

Similarly, therapies to delay or prevent onset of Type 1 Diabetes Mellitus (T1D) in high-risk populations would be extremely valuable but require identification and treatment of patients prior to disease onset. The Type 1 Diabetes Consortium (T1DC) has used an aggregated database of 2220 patients from three studies to investigate the use of pancreatic islet autoantibodies as enrichment biomarkers for clinical trials of therapies designed to delay or prevent onset of T1D. T1DC is developing a model to characterize the probability of T1D diagnosis over the course of a clinical trial, based on the presence of specific insulin autoantibodies and other patient features. This model is intended to allow drug developers to enrich prevention trials with patients at risk of developing T1D during the trial period, reducing the size and duration of trials required to see significant drug effects. The T1DC model was awarded a letter of support from EMA in 2020 [25], demonstrating the potential value and need for such a tool in clinical trials.

### Endpoint Selection

A major challenge in clinical trial design is selection of clinically meaningful endpoints, particularly in progressive diseases, where a single endpoint may not be appropriate at all stages of disease. For example, in Duchenne muscular dystrophy (DMD) endpoints may not be measurable or may only be sensitive to change in some of the trial population due to loss of abilities as disease progresses. As a result, interpretation of drug effects in this population is challenging [26, 27]. The Duchenne Regulatory Science Consortium (D-RSC) has aggregated patient-level data, including over 24,000 observations from over 1100 individuals with DMD from five clinical trial and six real-world data (RWD) sources, such as natural history studies and clinical registries. The consortium has used the aggregated data to develop six disease progression models that help align the selection of trial populations with appropriate clinical outcome assessment endpoints. The models also help inform the size and duration of a trial [28]. These models are currently seeking regulatory endorsement by FDA and EMA.

In therapeutic areas that require lengthy clinical trials, medical product development is disincentivized. For example, currently available immunosuppressive therapies (ISTs) for use in kidney transplant recipients perform well in the first year after kidney transplantation, however 10-year graft failure rates approach 50%, in some populations [29]. With placebo trials being unethical and good short-term outcomes with standard of care therapy, trials of novel ISTs must be large and/or lengthy to show meaningful differentiation from the current standard. The lack of validated surrogate endpoints for use in this population has led to development stagnation in this field [30]. The Transplant Therapeutics Consortium (TTC), a collaboration of transplantation societies, industry, academia, and FDA, is working to share and aggregate data to seek regulatory endorsement of a surrogate endpoint capable of predicting long-term outcomes from shorter-term measurements [31]. Regulatory endorsement of a surrogate endpoint would open accelerated approval pathways, previously unavailable in kidney transplantation, allowing sponsors to seek conditional marketing approval based on results from trials of reduced length. While longer-term confirmatory trials are still required, opening the accelerated approval pathway greatly improves the development landscape for developers and allows patients living with a kidney transplant timelier access to new therapies. The TTC database currently includes data from over 21,000 kidney transplant recipients from 10 clinical trials and 17 RWD clinical transplant center sources.

These examples highlight two cases where data from real-world sources were used, alongside data from controlled clinical trials, in the development of tools that will accelerate the product development process. RWD, defined as “data derived from sources other than traditional clinical trials,” represent an important source of information that, when shared and aggregated effectively, can help to generate the evidence necessary to inform regulatory decision-making during product development [32]. Other efforts that explore the usage, sharing, and aggregation of additional sources of RWD, such as electronic health record data, are ongoing.

### Stratification of Patient Populations

Rates of disease progression in amyotrophic lateral sclerosis (ALS) is highly variable, and clinical trials must therefore be inefficiently large to detect effects of a medical product. Prize for Life, a non-profit organization, leveraged a patient-level database to develop models that predict the rate of progression in ALS patients. Currently, the PRO-ACT database consists of 23 completed Phase 2 and 3 clinical trials and includes 10,723 records from individuals with ALS [33, 34]. Several groups used the PRO-ACT database to identify predictors of ALS progression rate and to develop predictive disease progression models. These tools allow developers to identify patients likely to progress rapidly, and therefore improve the likelihood of seeing effects from investigational agents. As a result, clinical trials can rely on fewer patients, reducing costs, while maintaining confidence in trial results.

### Clinical Trial Simulation

The drug development failure rate in Alzheimer's disease (AD), from 2002 through 2012, is reported to be 99.6% [35]. When promising agents fail Phase 3 trials after Phase 2 success, uncertainty often remains regarding the agents’ true effects. Failures of promising drugs may be a result of poorly designed clinical trials that are incapable of capturing drug effects. Clinical trial simulation (CTS) tools allow for in silico optimization of clinical trial design prior to trial initiation and can provide confidence by removing uncertainty in the interpretation of trial results. The Critical Path for Alzheimer’s Disease (CPAD), formerly the Coalition Against Major Diseases, partnered with FDA to lead an intensive data sharing initiative with a goal of delivering robust tools to facilitate better clinical trials for this condition. CPAD’s database currently includes data from over 41 sources, representing over 20,700 patients. The CPAD database led to the creation of the first regulatory-endorsed CTS tool, which is now publicly available for use. The CTS tool allows drug sponsors to choose various trial design scenarios, including subject enrollment criteria, trial duration, follow-up frequency, and estimated drug effects, then run simulated trials to predict outcomes. Simulated trials cannot replace live trials but allow sponsors to fine tune and streamline trial design before initiating live studies. As a result, trials can require fewer patients in the treatment or placebo arm, trial design being tailored to specific patient subtypes, and other potential benefits [36]. The complex disease-progression modeling underpinning the CTS tool requires a large volume of independent and heterogenous patient-level datasets, only feasible through data sharing and collaboration. The CTS tool has been endorsed by FDA and EMA, and to date has been used by more than 100 approved applicants. A similar CTS tool, which includes use of imaging biomarkers, is under development for pre-dementia trial enrichment. The pre-dementia CTS has received a letter of support from EMA and is currently undergoing endorsement review by FDA [37].

## Accelerating Therapies for COVID-19

Development of therapies for COVID-19 has occurred at an unprecedented rate, with the first therapeutics already approved by FDA through emergency use pathways. While early drug authorizations were based on standard clinical trial processes, public–private partnerships that aim to share data will further accelerate the development of future COVID-19 therapeutics. One such effort is the CURE Drug Repurposing Collaboratory (CDRC) [38]. Launched in June of 2020, CDRC aims to capture and aggregate shared RWD of off-label usage of existing medications for diseases of high unmet needs. This data is made publicly available and be used to inform future clinical trials and generate the necessary real-world evidence required to expand the labeling of these medications, include newly emerging infections, such as COVID-19. CDRC leverages data shared through multiple sources, including the CURE-ID application, existing patient registries, and directly from electronic health records. During the COVID-19 pandemic, CDRC has been digesting the data with the CURE-ID application through bioinformatic pipelines using artificial intelligence algorithms and natural language processing to identify potential signals for existing therapies that may be effective at treating COVID-19. While these signals alone may be insufficient to support a regulatory label expansion, they form the basis of real-world evidence required the advance drug repurposing for COVID-19. The CURE-ID application also links the FDA Adverse Event Reporting System to identifying potential safety concerns related to the use of potential COVID-19 therapies.

## Data Transparency Versus Data Sharing

Although successful data sharing has become more common and many organizations are working towards making data “FAIR” (Findable, Accessible, Interoperable, Reusable), patient-level data sharing still encounters many challenges, and most data sharing initiatives are insufficient to support regulatory innovation. An important distinction must be made between data transparency and actionable patient-level data sharing. Many companies have data transparency policies that require outside entities have access to view data, but often do not allow data to be transferred, downloaded, or otherwise physically copied to outside research spaces [39]. While data transparency is an indispensable aspect of proper industry stewardship, and can sometimes permit important data analyses, it typically does not enable data sharing capable of supporting product development tools. Many data transparency initiatives explicitly prohibit the transfer of data. These limitations are perceived as a necessary and important means to ease concerns from data owners and improve data sharing, but precludes the ability to aggregate data from multiple sources (including those that don’t participate in a specific data aggregation portal) into one integrated database and to transfer that data to regulatory authorities, as is required when seeking regulatory endorsement of a tool.

Data transparency portals often require data contributors and users to sign pre-written and non-negotiable data use agreements (DUAs) or data contribution agreements (DCAs), the legal documents that govern all aspects of the data sharing process. DUAs for many portals limit the types of allowable research and hinder the use of data from multiple sources to create a publicly-available, novel tools. DCAs from these portals are often inflexible and may not adequately alleviate major concerns from potential data contributors. Alternatively, using individually negotiated DCAs better facilitates data-sharing by addressing contributors’ specific concerns, while simultaneously maximizing the impact of data sharing by facilitating regulatory review of novel tools. This approach provides added flexibility to increase the likelihood of data sharing occurring, while supporting the use of that data to actionably impact medical product development.

## Common Concerns Regarding Data Contribution

Most concerns regarding data sharing fall within four general categories: data control and ownership, accessibility and acceptable usage, security, and patient privacy. In all cases, risk mitigation strategies exist, and nearly all perceived barriers have articulated and proven solutions (see Table 2).

**Table 2:** Common Barriers to Data Sharing

Stakeholder Group	Barrier	Mitigation
Industry	(1) Access to data could identify new safety or efficacy concerns (2) Competitors gaining access to data	• Limiting use cases or analyses of data • Sharing placebo or control arm data only • Limiting access or use cases for data
Academic researchers	(1) Data may be published ahead of primary researcher publication	• Restricting publication timing • Limiting or delaying access to outside researchers • Requiring joint authorship for publications • Right to review publications
	(2) Non-experts may mis-interpret data (3) Desire to charge industry for access to data to support ongoing data collection	• Right to review publications • Engaging in collaborative development of tool based on contributed data • Limiting use of integrated data by stakeholder group • Control of use of data through a process of review • Data contribution as in-kind contribution to PPP/consortium for specific uses
Patients/patient groups	(5) Ability to retain connection with industry	• Referring all database users to patient group • Engaging patient groups on use of data for tool development as part of PPP
	(6) Data protections	• Demonstrating data security solutions
	(7) Patient privacy	• Limiting sharing to de-identified data • Implementing policies to prevent re-identification

Concerns regarding control and ownership of transferred patient-level datasets are commonly seen from industry and academic researchers, as substantial financial investment can be required to generate high quality datasets. The perception that transferring data implies a loss of data ownership can lead potential data contributors to be reticent to share data. This concern is mitigated by explicitly stating ownership is retained by the data contributor in the DCA.

Potential data contributors may also be concerned that data may be accessed or used such that the data contributor’s individual interests could be harmed. Access to and acceptable uses of shared data can also be addressed through individually negotiated DCAs. These DCAs allow for each data contributor to determine a degree and time frame of data access and use commensurate with their concerns. For example, a DCA could require publications using contributed data occur after publication from the original contributor, or any publications based on contributor data be reviewed by the contributor prior to publication. Review would allow data custodians to refute conclusions that they feel are incorrect or misleading and ensure that the original data is appropriately referenced and cited.

In the case of sharing data to support medical product development tools intended to be submitted to regulatory agencies, at a minimum, DCAs must allow for data to be transferred to the convening organization, anonymously aggregated with other shared datasets, and securely transferred to regulatory authorities as part of regulatory submissions. Individual datasets must be accessible to the third-party convening organization, but, in nearly all cases, only the aggregated database must be accessible to regulatory authorities. Data contributors can elect, but are not required, to make their data more broadly accessible, for example to external qualified researchers. Specific access and use limitations can also be implemented for any dataset, such as limiting sharing to scenarios where contributed data comprises no more than a predefined percentage of the total aggregated database, or access only being granted to researchers following approval of a scientific review committee.

Data security and patient privacy are fundamental concerns when discussing data sharing of any kind. While all data sharing organizations have secure mechanisms for data transfer and security procedures in place, data sharing can be encouraged through a willingness to use an alternative, equally secure, transfer protocol preferred by the data contributor. Similarly, patient privacy must be respected and protected, and appropriate steps must be taken to ensure privacy is maintained, including assuring individual people cannot be identified in shared datasets. Steps should be taken to ensure patient consent for secondary use of data has been secured prior to sharing data. The DCA should also specifically address how data will securely transferred and patient privacy protections.

While rare, some scenarios exist where patient-level data sharing may be unfeasible. For example, historic datasets may only exist on paper in a storage facility. Similarly, industry mergers and acquisitions can result in decentralized data servers and loss of dataset knowledge or expertise. The resources required to locate, digitize, curate, and transfer this data are substantial and may outweigh the benefits of sharing. Fortunately, these scenarios are exceedingly and increasingly rare, and in most cases, perceived barriers to data sharing can be overcome through meaningful dialogue between the data contributor and data aggregator.

## Conclusions

Conceptually, data sharing is easy and straightforward and championed by all. In reality, meaningful and actionable data sharing resulting in tangible advances in product development is arduous and requires commitment from all stakeholders. When successful, the impact of data sharing is profound, resulting in tools that optimize drug development and help bring new therapies to patients in need. Sufficient case examples of successful data sharing are now available and should inform and encourage future data sharing initiatives. Further, the collaborative research environment fostered during the COVID-19 pandemic should be optimized, and lessons learned should be continued into the future to support accelerated progress across medical product development.

Successful patient-level data sharing will continue to require all stakeholders to work together to expand the precompetitive space. Those collecting data should agree to the use of standards and common data elements in the collection of data, and data owners must commit to sharing data and engaging in collaborative partnerships [40]. Organizations that convene PPPs and consortia must continue to provide safe, cooperative spaces for precompetitive data sharing and respond to concerns of potential data contributors. Regulatory agencies must continue to participate and encourage participation in these collaborative initiatives. When communities come together with a shared goal to collaborate, the profound impacts of patient-level data sharing are realized by individual organizations and, most importantly, patients. Reaffirming and expanding our commitments to effectively sharing data for the betterment of public health will result in dramatically improved medical product development and more rapid development of life-changing therapies for patients.
